# Applications of Human Amniotic Membrane for Tissue Engineering

**DOI:** 10.3390/membranes11060387

**Published:** 2021-05-25

**Authors:** Mathilde Fénelon, Sylvain Catros, Christophe Meyer, Jean-Christophe Fricain, Laurent Obert, Frédéric Auber, Aurélien Louvrier, Florelle Gindraux

**Affiliations:** 1 Université de Bordeaux, INSERM, BIOTIS, U1026, F-33000 Bordeaux, France; mathilde.fenelon@u-bordeaux.fr (M.F.); sylvain.catros@u-bordeaux.fr (S.C.); jean-christophe.fricain@inserm.fr (J.-C.F.); 2 Service de Chirurgie Orale, CHU Bordeaux, F-33076 Bordeaux, France; 3 Laboratoire de Nanomédecine, Imagerie, Thérapeutique EA 4662, Université Bourgogne Franche-Comté, F-25000 Besançon, France; c3meyer@chu-besancon.fr (C.M.); laurentobert@yahoo.fr (L.O.); fauber@chu-besancon.fr (F.A.); 4 Service de Chirurgie Maxillo-Faciale, Stomatologie et Odontologie Hospitalière, CHU Besançon, F-25000 Besançon, France; alouvrier@chu-besancon.fr; 5 Service de Chirurgie Orthopédique, Traumatologique et Plastique, CHU Besançon, F-25000 Besançon, France; 6 Service de Chirurgie Pédiatrique, CHU Besançon, F-25000 Besançon, France; 7 Inserm CIC 1431, CHU Besançon, F-25000 Besançon, France; 8 Université Bourgogne Franche-Comté, INSERM, EFS BFC, UMR1098, Interactions Hôte-Greffon-Tumeur/Ingénierie Cellulaire et Génique, LabEx LipSTIC, F-25000 Besançon, France

**Keywords:** amniotic membrane, cells, biological scaffold, tissue engineering, repair, reconstruction

## Abstract

An important component of tissue engineering (TE) is the supporting matrix upon which cells and tissues grow, also known as the scaffold. Scaffolds must easily integrate with host tissue and provide an excellent environment for cell growth and differentiation. Human amniotic membrane (hAM) is considered as a surgical waste without ethical issue, so it is a highly abundant, cost-effective, and readily available biomaterial. It has biocompatibility, low immunogenicity, adequate mechanical properties (permeability, stability, elasticity, flexibility, resorbability), and good cell adhesion. It exerts anti-inflammatory, antifibrotic, and antimutagenic properties and pain-relieving effects. It is also a source of growth factors, cytokines, and hAM cells with stem cell properties. This important source for scaffolding material has been widely studied and used in various areas of tissue repair: corneal repair, chronic wound treatment, genital reconstruction, tendon repair, microvascular reconstruction, nerve repair, and intraoral reconstruction. Depending on the targeted application, hAM has been used as a simple scaffold or seeded with various types of cells that are able to grow and differentiate. Thus, this natural biomaterial offers a wide range of applications in TE applications. Here, we review hAM properties as a biocompatible and degradable scaffold. Its use strategies (i.e., alone or combined with cells, cell seeding) and its degradation rate are also presented.

## 1. Introduction

Tissue engineering (TE) aims to induce tissue growth by combining cells, scaffolds, and growth factors or biomolecules [1]. Hence, the cells require the development of a scaffold from native or synthetic biomaterials, or a combination of the two, that mimics the extracellular matrix (ECM) [2]. The scaffold should have tissue integration properties and should be easily colonized with cells and be able to adhere, proliferate/survive, differentiate, and replicate the cell/tissue function [3]. The ideal scaffold requires easy handling and production, with biocompatibility, biodegradability, and mechanical properties consistent with the anatomical site of implantation. Depending on the application, it could be selectively permeable (to avoid invasion by fibrous tissue [4]) or porous (to ensure cellular penetration and adequate diffusion of nutrients to the cells within the construct and to the ECM formed by these cells [3]). When required, it should create and maintain space [4].

We have developed several TE products combining cells with biocompatible scaffolds [5,6]. We have been studying the benefit of the human amniotic membrane (hAM) in bone and nerve repair and in oral and maxillofacial surgery over the last few years [7,8,9,10,11,12,13,14,15,16,17,18,19,20,21,22,23,24].

The hAM derived from the placenta is considered surgical waste that can be obtained after elective cesarean surgery. It is thus a highly abundant, readily available, and cost-effective biological tissue that does not raise ethical issues. Thanks to its unique biological properties, this natural membrane has been used for over a century in medicine, especially in the field of ophthalmology and dermatology [25,26]. hAM is known to display several biological properties that are able to promote wound healing. It is a biocompatible immune-privileged tissue that exerts an anti-inflammatory, antifibrotic, antimicrobial, and antimutagenic effect [27,28]. hAM is a source of growth factors, cytokines, and hAM cells with stem cell properties [26,29,30]. Moreover, it combines adequate mechanical properties (permeability, stability, elasticity, flexibility, resorbability) [31,32] with good cell adhesion capacity, thanks to its natural ECM structural components (hyaluronic acid, collagens, laminin, fibronectin, and proteoglycans) [33].

**Figure 1:**
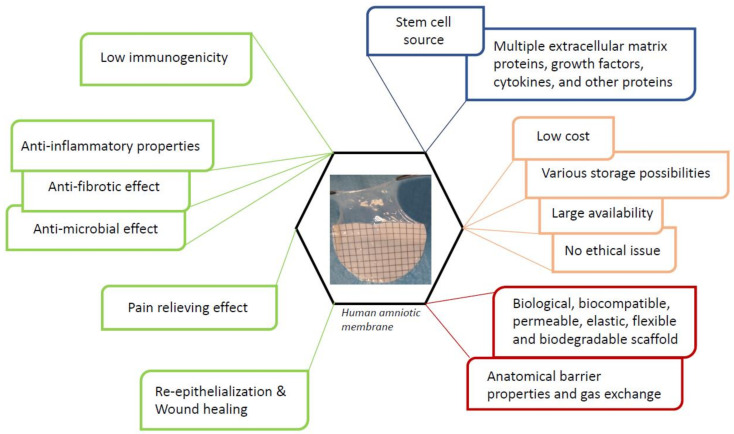
Human amniotic membrane properties as an ideal scaffold for tissue engineering.

Consequently, hAM represents a “ready to use” TE product, containing inherent cells and growth factors [34,35,36] (Figure 1). In addition, it is a suitable natural scaffold for cell seeding, proliferation, and/or differentiation. Thus, hAM-based scaffolds have been developed to improve its healing capacity and, mostly, to produce a qualified TE construct.

The efficacy of hAM alone or combined with cells has been widely investigated in experimental and clinical studies. Therefore, its support function is emphasized by the development of hAM composites and commercial products [9,31,32,36]. The use of hAM cells in the TE field is more sporadic [37].

The purpose of this review is to describe hAM properties as a biocompatible and degradable scaffold for TE applications. Moreover, an overview of hAM used alone or combined with cells is presented. We also aim to explore cell seeding and the degradation rate of hAM as there is currently limited data in the literature.

## 2. Human Amniotic Membrane

### 2.1. Anatomy and Physiology

**Figure 2:**
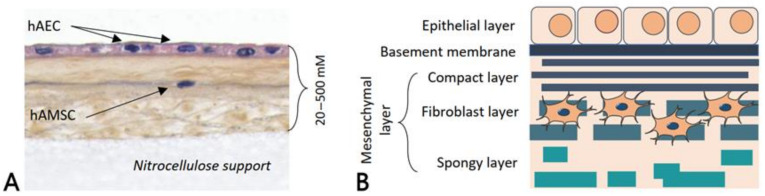
(**A**) Histological staining of fresh human amniotic membrane. hAEC: human amniotic epithelial cell, hAMSC: human amniotic mesenchymal stromal cell. (**B**) Representative structure of human amniotic membrane. The epithelial side, which consists of a monolayer of hAECs, and the mesenchymal layer, composed of hAMSC. A thick basement membrane separates both sides.

The human placenta plays a key role in the development and survival of the fetus, acting as physical and biological protection [38]. It is composed of two fetal membranes: an outer chorionic membrane and an inner hAM or amnion. hAM lines the amniotic cavity, in contact with the amniotic fluid. It contains three main layers: an epithelial monolayer that is separated from the stroma layer by a basement membrane (Figure 2) [26].

The amniotic epithelium is characterized by a single layer of human amniotic epithelial cells (hAECs), which usually have a columnar or cuboidal shape. It has been reported that hAECs express stem cell markers, retain the pluripotency of the undifferentiated epiblast, and have pluripotency and the ability to differentiate toward all three germ layers [29,30,39]. hAECs are densely adherent to the basement membrane, which lies at their outer edge. These cells secrete collagen type III and IV as well as noncollagenous glycoproteins (laminins, nidogen, and fibronectin) that form the basement membrane of the hAM [40]. This basement membrane is one of the thickest found in humans, and it provides support to the fetus during gestation. The third layer, called the stroma layer, is a collagen-rich mesenchymal layer that contains three components: (i) a compact layer, which is a dense and almost acellular layer mainly composed of collagen type I and III and fibronectin; (ii) a fibroblastic layer, where fibroblast-like mesenchymal cells (human amniotic mesenchymal stromal cells (hAMSC)) and rare macrophages with a loose fibroblast network can be observed; and (iii) the outer layer, called the spongy layer because of the high quantity of proteoglycans and glycoproteins leading to a spongy appearance on histological sections [38,41,42]. This spongy layer is made of loosely arranged collagen fibers and separates the amniotic and chorionic mesoderm. Collagen types I, III, V, and VI, secreted by the hAMSC, are the major proteins of the ECM in the stroma layer [43]. Those cells meet MSC minimal criteria [29], with divergence about their pluripotency [30]: hAMSC lack markers associated with pluripotency, such as TRA-1-60 and TRA-1-81 [44], whereas pluripotency markers SSEA-3 and SSEA-4 were reported to be positive [45].

hAM is a translucent biological structure that is neither vascularized nor innervated. Nutrients and oxygen are provided by the surrounding chorionic fluid, amniotic fluid, and the fetal surface vessels through diffusion mechanisms [34].

### 2.2. Collection and Preservation Methods

**Figure 3:**
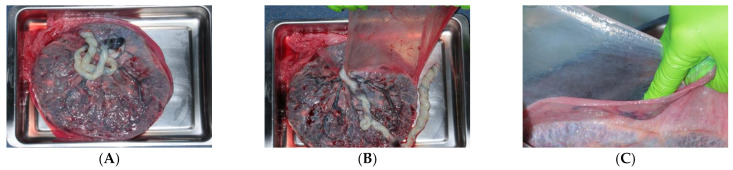
Human amniotic membrane collection. (**A**) Placenta. (**B**) Amnion and chorion. (**C**) Amnion detached from the chorion.

Placenta is generally obtained from healthy pregnant patients undergoing elective caesarian surgery after proper informed consent [26]. A rigorous serological screening must be performed on pregnant donors for human immunodeficiency virus-1/2, Hepatitis B, Hepatitis C, human T-cell lymphotropic virus, syphilis, cytomegalovirus, and tuberculosis. Placentas obtained from caesareans are the preferred source because placentas from vaginal deliveries can be contaminated and, therefore, unsuitable for transplantation. After the delivery, the collected placenta is placed in a sterile transport medium to avoid drying. Then the placenta is processed under aseptic conditions to obtain hAM. After repeated rinses of the placenta, the amnion is easily separated from the underlying chorion along their natural cleavage plane since the hAM spongy layer is loosely connected to the chorion (Figure 3). The placenta is routinely washed using a saline sterile solution containing antibiotics such as streptomycin, penicillin, neomycin, and amphotericin prior to storage [46].

Long-time storage before use is recommended by regulatory agencies of many countries to avoid the possibility that the donor is in the “window period” of infection. Thus, several preserving methods, such as cryopreservation, freeze-drying (lyophilization), or air-drying, have been developed. Whatever the method used, the processing and preservation of hAM will affect the properties of the biological material [47].

Cryopreservation in glycerol, acting as a cryoprotectant, is the most commonly used preservation method. Several studies reported the use of dimethyl sulfoxide as an alternative solution to cryopreserve hAM [48]. The cryopreserved format is safe and efficient, as reported by many experimental and clinical studies. Cryopreservation allows better preservation of proteins and growth factors compared to lyophilization, which is especially important when the tissue contains few proteins [49]. However, cryopreservation has some limitations and impacts the viability of the hAM cells [11,14,50]. Moreover, it requires expensive and cumbersome equipment to freeze a high quantity of amniotic tissue to −80 °C. Moreover, storage cannot exceed several months. Another difficulty is the necessary respect of the cold chain, making transportation difficult [46,51].

Lyophilization or freeze-drying is a preservation technique that consists of removing water from tissue by the process of sublimation. This process induces some alterations concerning structure, biological, and physical properties [49]. However, it results in a decrease of destructive chemical reactions, avoiding tissue deterioration, and the samples can be stored safely for several years at room temperature [49,51,52]. Transport is simple, in contrast to cryopreserved hAM [52]. A pretreatment with trehalose prior to lyophilization has been proposed to improve its quality. As the water loss caused by lyophilization may affect the physical and biological structures of amnion, trehalose can replace some water content in the cells, and it might have a positive effect on the stabilization of proteins and other components [46,53].

Air-drying is another preservation technique that is low-cost, and the final product is easy to store at room temperature [47,54]. hAM is kept at room temperature under a laminar flow hood and exposed to air for different time periods.

Lyophilization and air-drying are usually followed by sterilization of the hAM by gamma-radiation [48]. Sterilization with peracetic acid has also been proposed as an alternative to gamma-radiation [55]. Both products can be easily cut to the desired size and shaped with scissors just before use [56,57]. In addition, the graft is ready to use, contrary to glycerol-preserved membranes that require thawing and rinsing for approximately 1 h.

Several enzymatic, chemical, or mechanical techniques have been developed for denuding hAM [46]. Indeed, denuded (or de-epithelized) amnion promotes better cell proliferation and differentiation, better structural integrity, and more uniform cell outgrowth compared to the intact format [58,59]. Hence, it has been the preferred choice for ocular surface reconstruction. Similarly, decellularization treatment has also been applied to hAM. It aims to remove the major immunogenic cellular components, membrane-associated antigens, and soluble proteins, thus preventing the initiation of a cell-mediated or humoral immune response and subsequent degradation and rejection after clinical implantation [60,61]. Decellularization results in a significant decrease in hAM thickness without significantly decreasing its ultimate tensile strength, extensibility, or elasticity [60]. Both de-epithelization and decellularization strategies could be applied to fresh and cryo-preserved hAM. They are mainly combined with lyophilization or air-drying.

### 2.3. Biological Properties

hAM is an immune-privileged tissue as it contains some immunoregulatory factors, such as HLA-G, which is an immunosuppressive factor, and the Fas ligand [28]. This effect is also supported by the low/absent level of expression of HLA class I molecules and the absence of HLA class II molecules [62], thereby avoiding allograft rejection. Several growth factors are produced by hAM cells, such as epidermal growth factor (EGF), keratinocyte growth factor (KGF), hepatocyte growth factor (HGF), vascular endothelial growth factor (VEGF), platelet-derived growth factor (PDGF), basic fibroblast growth factor (bFGF), and macrophage colony-stimulating factor (M-CSF) [63,64]. Moreover, hAM has an anti-inflammatory effect, driven by both hAECs and hAMSC, which express various antiangiogenic and anti-inflammatory proteins such as the interleukin (IL)-1 receptor antagonist, tissue inhibitors of metalloproteinase (TIMPs)-1, -2, -3, -4, and IL-10 [65]. It has both angiogenic and antiangiogenic properties [66]. A few studies have suggested that hAM cells may exert an anticancer effect [67,68], mainly explained by the antiangiogenic, proapoptotic, and immunoregulatory activities of amnion.

hAM is also known to induce an antiadhesive and antiscarring effect. It reduces protease activity via the secretion of tissue inhibitors of TIMPs, and downregulates the expression of transforming growth factor beta (TGF-β), which is responsible for the activation of fibroblasts, thereby inducing an antifibrotic effect [27,69]. hAM is also known to exert an antimicrobial effect and, therefore, protects the wound from infection [70]. The antibacterial effect of hAM can be illustrated by its expression of natural antimicrobial molecules such as β-defensins and elafin [71] and its inhibitory effect against several bacteria (streptococcus group A or *S. aureus*) [72]. It can also be explained by its close adherence to the wound surface, avoiding contamination [73]. This close adherence is also known to maintain a moist environment, which contributes to the pain-relieving effect of hAM [48]. Indeed, it can be used to reduce the pain of burn or surgical wounds, acting as a biological dressing that protects the exposed nerve [74]. Finally, several studies have highlighted its ability to enhance re-epithelization [51,75].

Biological properties have to be modulated by the variability of hAM due to inter- and intradonor variations [76,77,78], subregional differences [79], or preservations methods [80], but this is without any clinical evidence.

### 2.4. Mechanical Properties

The physical properties of hAM, such as elasticity, stiffness, and mechanical strength, are other key elements of its attractiveness for TE [20,46]. Amnion is one of the thickest human membranes that adhere firmly to an exposed surface, for example, osteoarthritis articular cartilage [73]. Fresh hAM is a translucent tissue, and its thickness ranges from 0.02 to 0.5 mm. Collagens, elastin, and other ECM components play an important role in its biomechanical properties [43,81]. Indeed, it has been suggested that collagen proteins play a key role in the stress tolerance of fetal membranes because it has been observed that the collagen content was reduced in pathological fetal membranes that ruptured early [43]. Moreover, it seems that collagen types I and III predominate and form parallel bundles, providing the mechanical integrity of hAM. Collagen type V and VI form filamentous connections between interstitial collagens and the epithelial basement membrane [35]. To enhance its mechanical properties or to overcome the lack of space-maintenance capabilities, the use of multi-layered hAM [82,83] has been suggested or, alternatively, to reinforce hAM with a stronger biomaterial such as the electrospun nanofibers of polymers [84,85] or viscoelastic electrospun nanofibrous silk fibroin [86].

**Figure 4:**
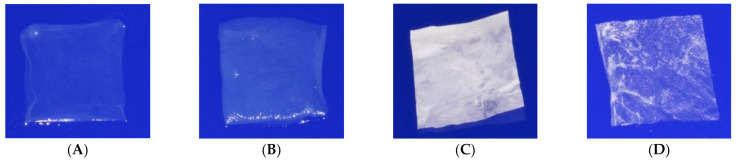
Human amniotic membrane formats. (**A**) Fresh. (**B**) Cryopreserved. (**C**) Lyophilized. (**D**) Decellularized and lyophilized [18].

Both physical and mechanical properties of hAM are also affected by preservation methods, sterilization, and cell removal (Figure 4) [18].

Cryopreservation often increases hAM’s thickness, whereas lyophilization decreases it [87]. Moreover, it was shown that cryopreservation did not affect some of hAM’s biomechanical properties [21]. Following rehydration, the lyophilized amnion returns to a layered structure; it thickens and becomes flaccid, and its transparency increases, suggesting that the membrane may have sufficient strength [56]. Recent studies have compared fresh, cryopreserved, lyophilized, and decellularized-then-lyophilized hAM [18,21]. In vivo, fresh hAM and decellularized-then-lyophilized hAM were significantly stronger than cryopreserved hAM and lyophilized hAM. Thus, the decellularization process increased the physical and mechanical properties of hAM. It made hAM significantly more stretchable than fresh hAM, significantly enhancing the tearing strength and significantly decreasing the hAM’s rate of resorption. One study also suggested that the sterilization process by gamma-radiation reduced its mechanical properties [88].

Moreover, differences in mechanical properties, thickness, and transparency have been reported depending on hAM subregions [20,76,79,89]. However, the clinical impact of such changes has not yet been evaluated.

### 2.5. Biocompatibility

Biocompatibility is the ability of a material to perform its desired function without causing any local or systemic adverse response in the recipient of the material [90]. As detailed before, hAM possesses a low risk of immunogenicity, which is an important criterion for a biocompatible scaffold [35]. Some authors have compared the in vivo biocompatibility of a synthetic scaffold and a biological scaffold made of hAM during the early phase of implantation in rats [91]. Histology and immunohistochemistry analyses revealed inflammatory infiltration in the synthetic-scaffold-implanted rats, but not in the hAM-implanted rats. At the same time, they demonstrated the in vivo biocompatibility of fresh hAM by complete blood count, clinical chemistry measurements, and immunohistochemical analysis.

hAM biocompatibility has also been evaluated following different preservation methods and/or osteodifferentiation [13,18,53,92,93]. It appears that fresh and preserved or osteodifferentiated hAM are biocompatible with slight variabilities, showing a slight-to-moderate inflammatory reaction compared to controls. In some applications, hAM has been used as a coating to improve the biocompatibility of other materials [32].

### 2.6. Cell Adhesion, Proliferation, and Differentiation

hAM has the ability to promote cell adhesion and proliferation, thanks to its ECM structural components (hyaluronic acid, collagens, laminin, fibronectin, and proteoglycans) [33]. Lyophilization improves its adhesion properties compared to fresh and cryopreserved hAM [87].

The application of hAM to the ocular surface results in an excellent substrate on which hAECs of the ocular surface can easily migrate, adhere, and grow [48]. That is why de-epithelization processes have been developed to expose the basal membrane in order to allow better cell proliferation and differentiation and the uniformity of cell outgrowth [58,59]. Zhang et al. compared the de-epithelialization of hAM by 20% ethanol, 1.2 U/mL dispase, 0.02% ethylenediaminetetraacetic (EDTA), 0.25% trypsin-EDTA, or 5 M urea, respectively, followed by gentle scraping [94]. The results indicated that urea denudation preserved basement membrane integrity, ECM, and growth factor composition and had higher cell attachment and proliferation efficiencies than the other modalities.

Four preparations were examined to determine the effect of total, partial, or non-decellularization on subsequent limbal epithelial cell expansion on hAM [55]. Complete removal of the hAECs resulted in a higher percentage of confluence of limbal epithelial cells but a lower cell density than the intact preparation. Thus, removing the hAM epithelium does not increase proliferation but, rather, facilitates migration of limbal epithelial cells that become larger in comparison with cell culture on intact amnion.

Fresh or preserved hAM, intact or denuded, and decellularized hAM have been used as biological substrates for cell culture growth with different cell types [35,87]. Moreover, the culture of human MSC on the hAM does not affect their immunophenotype or differentiation abilities [95]. Amnion was also used as a delivery system for chondrogenic MSC or adipose-derived MSC [96,97]. We investigated the capacity of fresh, cryopreserved, lyophilized, and decellularized-then-lyophilized hAM to support BM-MSC proliferation and osteodifferentiation [18,21]. We reported that decellularized format was the most suitable scaffold for BM-MSC proliferation and osteodifferentiation.

There are currently very few studies comparing the different sides of hAM to promote cell seeding. Initially, rabbit articular chondrocytes were seeded on three different hAM substrates: the epithelial side of intact hAM (IHE), basement side of denuded hAM (DHB), and stromal side of denuded hAM (DHS) [98]. While chondrocytes grew in a monolayer on the surface of the IHE and DHB substrates, the cells seeded in DHS penetrated and spread into the whole thickness of the stromal layer. The results suggested that denuded hAM was able to support chondrocyte proliferation with phenotype conservation in vitro and seemed more favorable when DHS was used. Later, Diaz et al. specified that the stromal side is more suitable than the epithelial one for human chondrocyte growth because of possible competition between chondrocytes and hAECs [73]. Both the basement membrane side and the collagenous stroma side of the acellular hAM matrix were capable of providing a preferential environment for driving the osteogenic differentiation of human dental apical papilla cells (APCs) with proven stem cell characteristics [99]. In addition, even without osteodifferentiation factors, APC cells differentiated on acellular amnion: more specifically, the collagenous stroma side was more effective than the basement membrane side.

Porcine urothelial cells were seeded on the hAM epithelium, denuded, and the stromal sides were cultured for 3 weeks [100]. The fastest growth and the highest differentiation of urothelial cells were demonstrated on the stromal version scaffold, which enabled the development of a tissue-engineered urothelium, with molecular and ultrastructural properties comparable to that of the native urothelium.

Recently, adipose-derived MSC and a human immortalized keratinocyte cell line (HaCaT) were seeded on the three different layers of the hAM and cultured for 3 weeks. Cell attachment and viability and the mechanical strengths of the basement membrane were assessed before and after cell culture [101]. All three layers supported the attachment and proliferation of cells with no visible cytotoxic effects. However, the growth and viability of both cell types cultured on the basement membrane were significantly higher than on the epithelial and stromal layers.

### 2.7. Biodegradation

Depending on the application/implantation site and species, hAM degradation varies from several days to several months [12,24], but the data are insufficiently reported. In ophthalmology, but also in wound healing, premature degradation may mean frequent repeat transplantations [24]. Exploiting hAM for corneal reconstruction, it has been observed occasionally that a residual subepithelial hAM may persist and inadvertently opacified the visual axis [26]. The fabrication of a composite material by adding silk to hAM in order to improve the degradation rate of hAM has been proposed [86].

Using a common murine animal model in a subcutaneous site, 8 weeks after implantation, all samples could be located. We noted a slight difference in tissue degradation between non-osteodifferentiated hAM (fresh hAM and cultured hAM) and osteodifferentiated hAM, probably due to a mineralized hAEC layer [13]. Additionally, we reported that the preservation methods of hAM may influence its degradation rate [18,21]. Decellularized-then-lyophilized hAM had the slowest rate of resorption compared to fresh, cryopreserved, and lyophilized hAM. It was the only membrane still present 8 weeks after subcutaneous implantation in rats.

## 3. Tissue Engineering Applications

Depending on the targeted TE application, hAM can be combined with natural or synthetic materials and/or additional cells. Here, a nonexhaustive list of publications was established for each tissue [31,32]. Examples of studies combining cells with hAM and no additional scaffold are summarized in Table 1. Clinical trials using hAM and/or hAM cells for regenerative medicine applications are reviewed in Table 2.

### 3.1. Eye

As mentioned earlier, hAM as a “simple scaffold” has a clinical indication in ophthalmology. Over the past two decades, excellent outcomes have been reported after transplantation of cultivated limbal stem cells on denuded hAM for limbal stem cell deficiency [102,103,104,105] or, similarly, with oral epithelium [106].

Later, different cell types were cultured on hAM to enhance its healing potential and expand its use to other indications in ophthalmology: it has resulted in several experimental and clinical studies (Table 1 and Table 2) [107].

As mentioned before, de-epithelialization and decellularization have been compared, with satisfying results, for limbal stem cell growth and/or migration [55,94].

A new method to fabricate a tissue-engineered corneal stromal in combination with keratocytes and multilayer ultrathin hAM was recently investigated [108]. A novel 3D biomimetic corneal model was developed to replicate corneal stromal organization with multilayer ultrathin hAM: it allowed the maturation of corneal stroma–like tissues in vitro.

In 2019, the first clinical study was conducted to evaluate the efficiency of using cultivated conjunctival epithelium transplantation on denuded hAM prepared using ice-cold urea as a basement membrane scaffold for cell-based tissue-engineered treatments of ocular surface disorders [109]. The protocol was applied to two patients, and the results indicated that this method could facilitate and mainstream a minimally invasive cell-based treatment for the reconstruction of extensive ocular surface wounds.

Monville et al. developed a human pluripotent stem cell retinal pigment epithelium sheet, disposed on hAM, that sustained the vision of rodents with retinal degeneration compared to the same cells injected in suspension [110]. After validation in a primate model [111], the first cell therapy for retinitis pigmentosa patients carrying retinal pigment epithelium gene mutations (LRAT, RPE65, and MERTK) was approved in 2019.

### 3.2. Skin

hAM has been used clinically for centuries as a biological dressing to treat acute and chronic wound injuries and burns, acting as a physical and biological barrier [112]. More than 200 clinical trials have reported its efficacy for wound healing [113].

A similarity between normal human skin and hAM layers exists. Consequently, amnion could provide a scaffold for a living-skin equivalent, greatly simplifying the procedures for making a dermal matrix and avoiding the use of animal collagen, which is costly and ethically problematic [74,114].

That is why, in addition to wound dressing, Yang et al. also suggested the use of hAM as a scaffold to create a skin substitute for wound closure. Amnion scaffolds seeded with human keratinocytes have generated living skin equivalents and have been successfully transplanted into an animal model [114]. Kim et al. recognized its added value in the management of full-thickness skin defects in rabbits [115]. Redondo et al. suggested the use of this allograft as a new strategy for inducing repigmentation in patients with vitiligo [116]. They cultured autologous melanocytes on a denuded hAM. The combined product was then implanted onto lesions of four patients with vitiligo, and the results showed a 90–95% repigmentation.

A new interesting and promising approach has been developed by Murphy et al. [113]. After grinding lyophilized hAM, they combined this solubilized allograft with hyaluronic acid, and they made a composite hydrogel delivery system. The aim was to obtain a cell-free solution while maintaining high concentrations of cell-derived cytokines and growth factors. This new amnion-derived material showed encouraging results to promote wound healing and reduce scar contraction in a full-thickness murine wound model.

### 3.3. Vascular System

Whereas cryopreservation is commonly used, Niknejad et al. suggested that lyophilized hAM is more suitable than the fresh and cryopreserved formats to culture endothelial cells [87].

The cell culture of porcine arterial endothelial cells on hAM has been proposed for the fabrication of tissue-engineered blood vessels [117,118]. They first demonstrated that porcine endothelial cells can successfully be seeded on sow’s hAM, with an increase in the expressions of junctional proteins while the expression of the adhesive inflammatory molecules decreases. Then, they realized tissue-engineered blood vessels made with rolled hAM, thus creating a tube of amnion that is endothelialized with porcine vascular endothelial cells [118]. The feasibility of a vein conduit fabrication from hAM and its implantation in the external jugular vein of juvenile sheep was also assessed [119].

An in vitro study reported the use of decellularized hAM seeded with human umbilical vein endothelial cells and human vascular smooth muscle cells prior to being rolled into a dense construct as an alternative strategy to develop cell-dense vascular bioscaffolds. It resulted in a mechanically stable, multilayered tissue-engineered blood vessel conduit that can be manufactured into different diameters and shapes to suit the targeted applications [120]. The acellular hAM conduits were surgically implanted as arterial interposition grafts into the carotid arteries of immunocompetent rabbits [121]. The grafts demonstrated patency over four weeks (n = 3), with no hyperacute rejection or thrombotic occlusion. Swim et al. combined decellularization and freeze-drying to produce a monolayer or a multilayer amnion-based scaffold suitable for TE constructs, designed for reconstructive heart surgery [122]. Whereas both preservation procedures enhanced the cell viability and growth of various cell types seeded on hAM in vitro, the multilayered construct displayed enhanced biomechanical properties. It was implanted in a piglet model of left pulmonary artery grafting. The results showed its in vivo suitability and biocompatibility for vascular repair, as demonstrated by the development of newly formed endothelium in the intima, a smooth muscle cell-rich medial layer and an adventia containing new vasa vasorum, an endothelial cell layer in the inner side of the graft, and a smooth muscle layer in the outer side [122].

Finally, an in vitro study evaluated the blood compatibility of the epithelial and stromal surfaces of the amnion for potential use in vascular TE [123]. These results suggested that hAM, which contains hAECs and hAMSC, has appropriate hemocompatibility to be employed in the field, especially as a vein substitute. No significant difference was seen between the epithelial and stromal sides of the amnion.

### 3.4. Bladder and Vagina

In the early 1980s, the first studies on animals with glutaraldehyde-stabilized or fresh hAM used for bladder reconstruction were reported [124], with fast epithelialization and improved functionality [125]. Later, it was observed that hAM may be a substitute for the transitional epithelium of the bladder in dogs [126]. Three layers of rehydrated hAM, sutured to the bladder defect, have been experimented on in vesico-vaginal fistulae, demonstrating a structured implementation of a new method for vesico-vaginal fistulae repair following IDEAL recommendations [127]. In an original way, a sandwich-structured biocomposite material was made of cryopreserved hAM, covered on both sides with two-layered membranes of electrospun poly-(l-lactide-co-ecaprolactone) for bladder augmentation in a rat model [85].

In a clinic setting, Brandt et al. explored the use of hAM grafts in 8 female patients with urological congenital defects. They reported that the procedure was quick and effective for appropriate restoration of the function and cosmetics of the lower urogenital tract [128].

Several authors have reported the use of hAM for vaginoplasty in patients suffering from congenital absence of the vagina or for gender reassignment surgery. The creation of a neovagina is, thus, associated with an amnion graft. Both fresh and preserved hAM were assessed and resulted in adequate anatomic and functional outcomes [129,130,131]. In vitro studies have investigated the growth pattern, morphology, and specific features of human bladder smooth muscle cells on two different matrixes, amnion and collagen, and showed abundant cell-to-cell adhesions with hAM [132]. Satisfactory outcomes were also obtained when autologous fibroblasts were seeded onto hAM prior to its graft to cover the neovagina [133]. The two layers of amnion and fibroblasts were more resistant to trauma and laceration than amnion without seeded cells.

### 3.5. Urethral

Based on the number of surveys conducted or ongoing clinical studies, urology has also played a large part in studies using hAM [84,134,135]. Shakeri et al. evaluated hAM as a xenograft for urethroplasty in rabbits [136]. They concluded that it was an inexpensive, simple, and biodegradable graft, yielding very little antigen effect, and a viable option in surgical urethroplasty. A study compared the effects of acellular hAM to synthetic poly-l-lactide-co-1-caprolactone on human urothelial cell viability, proliferation, and urothelial differentiation levels, with unfavorable results for the amnion [137]. Salehipour et al. evaluated its use in the reconstruction of long ureteral defects in dogs and speculated its efficacy as a patch graft versus a full circumferential graft in the reconstruction of ureteral defects [138].

In a clinical setting, Koziak et al. explored its use in the reconstruction of long ureteral structures in 2 and then 11 patients [139,140]. hAM was successfully used to supplement ureteral wall defects. Indications for the procedure included ureteral strictures of a 5.5 cm average (range, 3–8 cm), localized in different parts of the ureter: upper (5), middle (5), and lower (3).

The proliferation quality of mouse urothelial cells has been assessed on three natural matrixes of hAM, peritoneum, and omentum compared to collagen matrix, with promising results for the amnion [141]. As described before, the fastest growth and highest differentiations of urothelial cells were demonstrated on the hAM stromal side [100]. Denuded hAM, inoculated with primary rabbit urethral epithelial cells and applied as urethroplastic material in the rabbit models of urethral injury, displayed good biocompatibility [142]. Chen et al. seeded allogeneic BM-MSC and/or endothelial progenitor cells on decellularized amnion (with the cell-seeded surface facing the corpus spongiosum) as a treatment for urethral defects in dogs [143]. Subsequently, they concluded that hAM seeded with allogeneic endothelial progenitor cells +/− BM-MSC can more effectively repair a 3-cm circumferential urethral defect in a large animal model.

### 3.6. Cartilage

Similar components (hyaluronan acid, proteoglycans, and collagen) have been found between the ECM of hAM and native cartilage [33]. The potential of fresh, cryopreserved, lyophilized, or dried amnion to act as MSC [96,144] or chondrocyte [145] cell carriers and promote MSC chondrogenic differentiation was investigated with success.

As mentioned before, in vitro and in vivo studies performed on cryopreserved intact or de-epithelialized hAM have stated that its stromal side is a more suitable scaffold than its epithelial side to promote chondrocyte proliferation and to maintain their phenotype [73,98]. In vivo, denuded amnion alone was compared to denuded amnion seeded with chondrocytes to repair a rabbit osteochondral defect. The rate of regenerated cartilage was significantly higher when chondrocytes seeded on hAM were facing the defect, suggesting that denuded amnion can act as a cell carrier matrix for cartilage regeneration [98]. In vivo results suggested that fresh and cryopreserved amnions alone compared to cryopreserved amnion previously cultivated with BM-MSC showed similar regenerative properties [144].

Interestingly, hAM was combined with fibrin to develop a new 3D scaffold that was able to promote bovine chondrocytes in vitro proliferation [146]. Similarly, hAM has also been combined—as cell-free material—with a synthetic scaffold in poly-d,l-lactic-co-glycolic acid, which, once implanted in osteochondral defects, was able to promote regeneration of hyaline-like cartilage [147].

### 3.7. Bone

The ability of hAM to be osteodifferentiated in toto has been established in vitro [10,13,15,19,148]. However, when associated with a bone substitute and implanted in a mice subcutaneous model, fresh and in vitro osteodifferentiated hAM were not able to induce ectopic bone formation [13].

Interestingly, in an orthotopic model, fresh hAM had a periosteum-like effect when implanted over a segmental defect in rabbits [149]. Moreover, cryopreserved hAM slightly enhanced bone regeneration when used as a membrane for guided bone regeneration (GBR) in a murine calvaria model [14]. GBR function has also been explored with a decellularized–lyophilized format [21,150].

hAM showed similarities with the induced membrane (IM) [12]. The IM technique (also called the Masquelet technique) is a commonly used two-step procedure to treat segmental long bone defects. The first step allows the generation of a foreign body membrane (the IM), which protects the bone auto- or allograft from resorption by the local environment [151,152,153,154]. The similarity between these two membranes (hAM and IM) generated by the body may simplify the Masquelet technique into a single procedure, avoiding the time required for the formation of the IM and the second surgery. Accordingly, a decellularized–lyophilized hAM, as an alternative to the induced membrane technique, was used in a segmental femoral defect model [23].

Processed hAM, seeded with bone marrow (BM) or adipose-derived MSC, led to encouraging results in a calvarial bone defect animal model [155,156] and was a suitable scaffold for cell proliferation and osteogenic differentiation [18,21,150]. As shown before, even in the absence of osteoinduction, acellular hAM matrix exerted the substrate-induced effect of initiating APC differentiation [99]. In an original way, transplantation of MSC from periodontal ligaments and osteoblasts using double-layered cell transfer significantly enhanced in vivo bone formation compared to single-cell-type transplantation [157].

A clinical study reported the successful use of decellularized hAM in combination with autologous buccal-fat-pad-derived stem cells to treat large bone defects in jaws [158]. In the case series, the combination of bone substitutes (hydroxyapatite and platelet-rich fibrin) with amnion allowed periapical bone healing [159].

### 3.8. Oral, Periodontal, and Maxillofacial Surgery

Since its first use in 1985 by Lawson et al. for the treatment of oral mucosa defects [160], this allograft has been widely studied in the field of oral and maxillofacial surgery, and promising results exist for oral soft tissue regeneration [16]. Multilayered hAM was used to close oronasal fistula in minipigs [161] and in four patients [83]. Moreover, the amniochorionic membranes were compared to the conventional membrane already used for GBR procedures in oral surgery [162].

Several studies have reported the ability of hAM to stimulate healing and enhance epithelial regeneration of human oral mucosa defects after excision of benign and precancerous lesions [163,164]. In this context, a bioartificial mucosa using cultured oral keratinocytes on hAM was fabricated to evaluate the possibility of developing a prelaminated myomucosal flap using the fabricated bioartificial mucosa and a local muscle flap [165]. Similarly, denuded (hyper)dry or cryopreserved hAM have been used alone [56] or seeded with oral mucosal epithelial cell sheets and transferred to the mucosal defect in both preclinical [166,167] and clinical studies [168,169].

The use of hAM to treat root exposure caused by gingival recession has been successfully reported in several clinical studies. When the hAM graft was associated with a gingival flap, the root coverage and gingival thickness and biotype were improved [170,171,172].

In a combined way, autologous keratinocytes cultured on hAM combined with poly(L-lactic acid) were transplanted with success to cover intraoral fistulas and bone loss after osteoradionecrosis in 9 patients (15 procedures) [173].

Periodontal disease affects the supportive tissue of teeth, which include the periodontal ligament. Several preclinical studies have aimed to investigate the efficacy of hAM to treat periodontal disease. Amnion was seeded with periodontal ligament stem cells or periosteum-derived cells [166,174,175] or adipose-derived MSC [176]. These studies concluded that hAM could be a useful scaffold for periodontal regeneration by avoiding the proliferation of connective tissue on the denuded root surface in the periodontal defect. Finally, an in vitro study suggested the potential of dental-pulp-derived cell sheets cultured on hAM substrates for periodontal TE [177].

In maxillofacial surgery, hAM was used as an interpositional material to prevent temporomandibular joint re-ankylosis in a rabbit model [178]. Similar results were observed when cryopreserved hAM was compared to fresh hAM [179]. Improvement in chewing efficiency and the absence of pain were related in one case report [180]. Its use has also been tested with success in the treatment of two patients with bisphosphonate-related osteonecrosis of the jaw [181].

### 3.9. Nerve

Several studies have reported the use of fresh or preserved hAM as a scaffold for nerve regeneration, highlighting a proregenerative effect on injured peripheral nerves, thanks to its antifibrotic and antiscarring effects [17]. For example, fresh hAM was implanted in a rat model of sciatic nerve scarring to treat recurring perineural adhesions and the associated nerve scarring. Accelerated recovery of sciatic nerve function was observed when the epithelial side of hAM was applied toward the nerve [182]. To manage nerve injury, cryopreserved hAM was wrapped around the damaged nerve, and scar formation and functional recovery were assessed. Although both functional and morphological parameters were not significantly improved, the nerves wrapped with hAM had significantly fewer adhesions and less scar formation than controls [183]. For both indications, only a few studies have specified the orientation of the applied amnion [17]: stromal side against the nerve [184] or epithelial side [182,185]. In a combined way, a dehydrated amnion filled with skeletal muscle cells, harvested from neighboring tissue, showed encouraging results in humans for repairing post-traumatic nerve defects of 3 to 5 cm in length [186]. Later, this clinical proof of concept was substantiated by an experimental model [185]. Additionally, amnion tubes were manufactured to cover the gap and edges of the nerve with favorable functional in vivo results [187,188,189]. Recently, an electrospun polycaprolactone–amnion nanofibrous membrane showed satisfying results for the treatment of sciatic nerve compression in a rat model [190].

Only two articles were found with a TE purpose. The first reports the use of a scroll of amnion derivative (ZQ membrane) combined with cultured autogenous Schwann cells [191]. The second was the application of human umbilical cord MSC-loaded hAM for the repair of radial nerve injury, with functional recovery better in the transplantation group than the control group [192].

### 3.10. Ligament and Tendon

The interest in hAM for ligament and tendon healing has also been explored [193,194]. This tissue has the ability to prevent tendon adhesions after injury and reconstruction [195]. One study investigated the effects of fresh denuded amnion and hyaluronic acid, alone and in combination, on adhesions and healing following chicken flexor tendon repair. The prevention of adhesion formation was superior when hAM was wrapped around the repaired tendon [196]. Another study reported the effectiveness of decellularized amnion to promote endogenous healing and prevent tendon adhesion in the same model [197].

This method of tendon-wrapping, in which cryopreserved hAM is laid over the damaged tendon, has also been successfully reported in humans [198,199]. The addition of a de-epithelialized and lyophilized hAM wrap around a composite silk scaffold that included tenocytes accelerated cellular migration and angiogenesis in neotendons in rabbits [200].

We have found only one article with a TE purpose. It evaluated fetal skin fibroblast cells seeded on hAM ECM in an in vivo model of Achille tendon defect, with promising functional results [201].

### 3.11. Heart

In the field of cardiology, hAM patching improved ischemic heart repair in rat and mice models [202,203,204]. Similarly, an acellular hAM was explored in vivo as a pericardial substitute [205]. Results showed that hAM use increased the pericardium repair thickness, thanks to its ability to reduce the incidence of adhesions and scarring. Four weeks after surgery, host cells organized with tissue fibrils and capillaries were clearly identified in the surface (epicardial) coating of the hAM patch, indicating that its outer layer became well integrated with the host tissues.

Medical case reports have also stated that hAM patching had anti-inflammatory effects and reduced new-onset postoperative fibrillation in patients undergoing cardiac surgery [206,207].

In a combined way, a novel composite biomaterial was developed by processing human cardiac ECM into a hydrogel and combining it with cell-free hAM via a dry-coating procedure [208]. The researchers concluded that the incorporation of human cardiac ECM hydrogel shifts and enhances the bioactivity of decellularized hAM, facilitating its use in future cardiac applications. Overall, based on their results, this scaffold may be a potential platform for the epicardial delivery of cells and therapeutic agents as it possesses superior adhesion capacity, supports cell proliferation and viability, and modulates inflammatory responses.

Only one article was reported with a TE purpose. It investigated an in-house generated human-induced pluripotent-stem cell-derived cardiac progenitor seeded on trypsinized and cryopreserved hAM to construct a cardiac cell sheet [209]. The study showed that the seeded progenitor cells grafted onto the matrix of hAM differentiated in situ into functional and relatively mature cardiomyocytes. hAM slightly improved the development of cardiomyocytes compared to the control basement membrane matrix, Matrigel™.

### 3.12. Clinical Trials

Due to historical use and banking facilities, to date, hAM is mainly exploited under its scaffold format instead of its derived cells.

Consequently, the assessment of the overall number of clinical trials evaluating hAM as a scaffold for TE purposes revealed that ophthalmology has the biggest share, with 14 clinical trials (Table 2). In this indication, various autologous or allogenic cells have been seeded on hAM: limbal (epithelial) stem cells, cornea stem cells, oral mucosal epithelial cells, conjunctival epithelial cells, fibroblasts, and BM-MSC.

hAM-based scaffolds have been studied sporadically in Asherman’s Syndrome, endometrium infertile patients, and anterior cruciate ligament ruptures. Interestingly, in gynecology, hAM was combined with isolated hAECs. In this indication, these cells have also been explored alone in other clinical trials not included in Table 2 (NCT03207412, NCT02912104, NCT03381807, NCT03223454). Finally, both hAM cells and hAECs have been examined alone in wound healing and nonunion fracture, respectively.

**Table 2:** Clinical trials using human amniotic membrane cells and/or human amniotic membrane as a scaffold for tissue engineering purposes (https://clinicaltrials.gov (accessed on 7 October 2020)).

Conditions	Clinical Trials Id	Phase	Tissue Engineering Product Evaluated	Status	Sponsor	Results/Status or Remarks
OCULAR SURFACE DISEASE	NCT00348114	2	Amnion + ex vivo expanded limbal epithelial stem cells	Suspended	Singapore National Eye Centre	Estimated Enrolment: 8 participants Estimated Study Completion Date: May 2006
LIMBAL STEM CELL DEFICIENCY	NCT00736307	1 2	Amnion + cultured limbal epithelial stem cells	Completed	Royan Institute, Tehran, Iran	Enrolment: 10 participants Study Completion Date: October 2009
UNILATERALLIMBAL STEM CELL INSUFFICIENCY	NCT01377311	1	Amnion + cultured limbal stem cells	Terminated	National Taiwan University Hospital	Enrolment: 0 participants Study Completion Date: April 2010
LIMBAL INSUFFICIENCY SYMBLEPHARON	NCT00491959	1	Amnion + oral mucosal epithelial cells	Terminated (Due to unstable cell sheet quality, the technique was not tested on patients)	National Taiwan University Hospital	Enrolment: 0 participants Study Completion Date: April 2010
SYMBLEPHARON	NCT00799526	1	Amnion + ex vivo cultivated autologous conjunctival epithelial cells	Unknown	Federal University of São Paulo	Estimated Enrolment: 10 participants Estimated Study Completion Date: November 2010
EYE INJURY	NCT01123044	3	Amnion + autologous limbal epithelial cells	Unknown	Ministry of Health, Malaysia	Enrolment: 42 participants Estimated Study Completion Date: September 2012
EPIDERMOLYSIS BULLOSA WITH MITTEN HANDS	NCT01908088	1	Amnion + autologous fibroblasts	Completed	Royan Institute	Enrolment: 6 participants Study Completion Date: July 2013
CORNEAL DISEASE PTERYGIUM MYOPIA HYPEROPIA	NCT02148016	1 2	Autologous limbal stem cell + amnion as a protective contact lens	Unknown	Sun Yat-sen University	Estimated Enrolment: 30 participants Estimated Study Completion Date: September 2014
LIMBUS CORNEAE INSUFFICIENCY SYNDROME	NCT01562002	1 2	Amnion + allogenic bone marrow MSC versus amnion + allogenic limbal stem cells	Completed	Instituto Universitario de Oftalmobiología Aplicada (Institute of Applied Ophthalmobiology)—IOBA	Enrolment: 27 participants Study Completion Date: December 2014
OCULAR SURFACE RECONSTRUCTION	NCT01341223	Observational	Amnion as a carrier for ex vivo cell culture	Unknown	National Taiwan University Hospital	Estimated Enrolment: 50 participants Estimated Study Completion Date: Mars 2016
LIMBAL STEM CELL DEFICIENCY	NCT03226015	Observational	Amnion + autologous oral mucosa	Completed	Klinikum Chemnitz gGmbH	Enrolment: 27 participants Study Completion Date: May 2017
LIMBAL STEM CELL DEFICIENCY	NCT01619189	2	Amnion + allogeneic or autologous limbal epithelial stem cells	Completed	CHNO des quinze-vingtsParis, France	Enrollment: 14 participants Study Completion Date: 6 March 2017
LIMBAL STEM CELL DEFICIENCY	NCT02579993	Interventional	Amnion + in vitro expanded limbal stem cells	Terminated (Preliminary results not favorable)	Instituto de Oftalmologia Conde de Valenciana	Enrolment: 10 participants Study Completion Date: March 2018
LIMBAL STEM CELL DEFICIENCY	NCT02592330	1	Amnion + expanded autologous limbal epithelial cells	Recruiting	Massachusetts Eye and Ear Infirmary	Estimated Enrollment: 17 participants Estimated Study Completion Date: 30 June 2023
WOUNDS	NCT02314416	4	Amniotic stem cells + collagen matrix	Withdrawn	Augusta University	Enrolment: 0 participant Study Completion Date: May 2015
ASHERMAN’S SYNDROME	NCT03223454	1	Amnion + AEC	Unknown	The Second Affiliated Hospital of Chongqing Medical University	Estimated Enrolment: 50 participants Estimated Study Completion Date: March 2021
ENDOMETRIUM INFERTILE PATIENTS	NCT04676269	1	Amnion + autologous endometrium cells or allogenic AEC or both type of cells	Recruiting	Indonesia University	Estimated Enrolment: 40 participants Estimated Study Completion Date: 15 December 2021
ANTERIOR CRUCIATE LIGAMENT RUPTURE	NCT03294759	Interventional	Amnion collagen matrix wrap + bone MSC	Active, not recruiting	Andrews Research & Education Foundation	Actual Enrolment: 40 participants Estimated Study Completion Date: 25 September 2021
ANTERIOR CRUCIATE LIGAMENT RUPTURE	NCT03294720	Interventional	Amnion collagen matrix wrap + bone MSC	Active, not recruiting	Andrews Research & Education Foundation	Actual Enrolment: 10 participants Estimated Study Completion Date: 20 March 2021
NONUNION FRACTURE	NCT03031509	1 2	AEC	Not yet recruiting	Shanghai East Hospital	Estimated Enrollment: 36 participants Estimated Study Completion Date: December 2020

MSC = Mesenchymal Stromal Cells; AEC = Amniotic epithelial cells.

## 4. Conclusion

Since promising results have been achieved with hAM in ophthalmology and dermatology, an increasing number of publications have suggested its potential for other TE applications. Experimental works have described promising results in vascular, bone, and cartilage repair and oral surgery, similarly to the research conducted in overall TE in recent years. From our analysis, nerve, ligament, tendon, and cardiac applications were sporadic compared to urology.

In clinics, bone, oral mucosa, and ligament repair have been investigated, and two industrial clinical trials have been conducted. Thus, the participation of the industry in the TE field is highly anticipated. Some exogenous indications in gynecology (Asherman’s Syndrome and endometrium infertile patients) that do not properly belong to the TE field have also been explored.

Looking at the TE constructs more in detail, decellularization or, mainly, the de-epithelialization process has been applied to hAM. Predominantly in the cartilage TE area, researchers have compared the efficacy of seeding the three layers (epithelial, basement membrane, and stromal). The basement membrane layer seems to be more favorable for cell seeding, proliferation, and differentiation. To date, there is no consensus on the best cells to seed on hAM, and the choice is mostly driven by the tissue to regenerate. In all cases, a good balance must be found between a noninvasive procedure for the collection of cells and their final functional capacity. That is why, for example, oral mucosa has been considered a source of epithelial cells in ophthalmology. The in vivo degradation rate of hAM is not detailed enough in the literature and should be more evaluated.

Finally, we note that clinical trials have intensively explored hAM as a scaffold compared to the use of its cells as a TE construct. Increased knowledge of hAM cells, in particular regarding their function, will encourage future clinical investigations.

## Figures and Tables

**Table 1:** Use of amniotic membrane as a scaffold for tissue engineering.

Authors	Tissue Engineering Applications	Amniotic Membrane Formats	Modalities of Amniotic Membrane Usage	Cells Seeded on Amniotic Membrane	Sides of Cells Seeding	Assessment
Shortt et al., 2009	Ocular surface	Cryopreserved or Decellularized + Cryopreserved	Single membrane	Human limbal epithelial stem cells	Basement membrane (?)	In vitro/Ex vivo
Zhang et al., 2013	Ocular surface	Cryopreserved or De-epithelialized	Single membrane	Human limbal epithelial cells	Basement membrane	In vitro/Ex vivo
Che et al., 2019	Ocular surface	De-epithelialized	Multilayer ultrathin amnion (3–4 layers)	Human corneal stromal cells Keratocytes	Basement membrane Cells between the layers	In vitro/Ex vivo
Bandeira et al., 2019	Ocular surface	Cryopreserved + De-epithelialized	Single membrane/Cover	Human conjunctival epithelial cells	Basement membrane	Clinical study
Yang et al., 2006	Skin	Cryopreserved + De-epithelialized	Single membrane/Cover	Human keratinocytes	Basement membrane	In vitro/Ex vivo + In vivo
Kim et al., 2008	Skin	Cryopreserved + De-epithelialized	Single membrane/Cover	Rabbit bone marrow autologous or allologous MSC	Basement membrane	In vivo
Redondo et al., 2011	Skin	Cryopreserved + De-epithelialized	Single membrane/Cover	Human melanocytes	Basement membrane	Clinical study
Tsai et al., 2007	Vascular system	Cryopreserved + De-epithelialized sow amnion	Single membrane	Porcine vascular endothelial cells	Basement membrane	In vitro/Ex vivo
Niknejad et al., 2011	Vascular system	Fresh or Cryopreserved or Lyophilized	Single membrane	Rat vascular endothelial cells	Epithelial	In vitro/Ex vivo
Lee et al., 2012	Vascular system	Air-dried + De-epithelialized + Glutaraldehyde	Tube of amnion	Porcine vascular endothelial cells	NS	In vitro/Ex vivo
Amensag et al., 2012	Vascular system	Two cycles of freezing and thawing + Decellularized	Tube of six-layered amnion	Human umbilical vein endothelial cells Human vascular smooth muscle cells	Stromal	In vitro/Ex vivo
Amensag et al., 2017	Vascular system	Two cycles of freezing and thawing + Decellularized	Tube of six-layered amnion	Human vascular smooth muscle cells	NS	In vitro/Ex vivo + In vivo
Swim et al., 2018	Vascular system	Decellularized + Lyophilized	Multilayer amnion/Cover	Human thymus-derived MSC Human umbilical cord blood MSC Human umbilical vein endothelial cells Cardiac myocytes Arterial smooth muscle cells	NS	In vitro/Ex vivo + In vivo
Sharifiaghdas et al., 2009	Vaginal and bladder	Fresh + De-epithelialized	Single membrane	Human bladder smooth muscle cells	Basement membrane	In vitro/Ex vivo
Seyed-Forootan et al., 2018	Vaginal and bladder	Fresh	Two layers of amnion/Cover	Autologous skin fibroblasts	NS	Clinical study
Sharifiaghdas et al., 2007	Urethra	Fresh + De-epithelialized	Single membrane	Mouse urothelial cells	Basement membrane	In vitro/Ex vivo
Sartoneva et al., 2011	Urethra	Fresh + De-epithelialized	Amnion attached to a membrane fixation device (cell crowns)	Human urothelial cell	NS	In vitro/Ex vivo
Jerman et al., 2014	Urethra	Cryopreserved	Single membrane	Porcine urethral cells	Epithelial or Basement membrane or Stromal	In vitro/Ex vivo
Wang et al., 2014	Urethra	De-epithelialized	Single membrane/Cover	Rabbit urethral epithelial cells	NS	In vitro/Ex vivo + In vivo
Chen et al., 2018	Urethra	Decellularized + Lyophilized	Tube of amnion	Allogenic canine endothelial progenitor cells +/− bone marrow MSC	NS	In vitro/Ex vivo + In vivo
Jin et al., 2007	Cartilage	Cryopreserved or Cryopreserved + De-epithelialized	Single membrane/Cover	Rabbit chondrocytes	Epithelial or Basement membrane or Stromal	In vitro/Ex vivo + In vivo
Díaz-Prado et al., 2010	Cartilage	Cryopreserved or Cryopreserved + De-epithelialized	Single membrane	Human chondrocytes	Epithelial or Basement membrane or Stromal	In vitro/Ex vivo
Krishnamurithy et al., 2011	Cartilage	Air-dried or Lyophilized	Single membrane	Rabbit chondrocytes	Basement membrane	In vitro/Ex vivo
Tan et al., 2011	Cartilage	Air-dried or Lyophilized	Single membrane	Rabbit bone marrow MSC	NS	In vitro/Ex vivo
Garcia et al., 2015	Cartilage	Fresh or cryopreserved or and cryopreserved	Single membrane/Cover	Sheep bone marrow MSC	Stromal	In vitro/Ex vivo + In vivo
Tsugawa et al., 2011	Bone	Cryopreserved + De-epithelialized	Single membrane/Cover	Mouse bone marrow-derived osteoblast cells	Stromal	In vitro/Ex vivo + In vivo
Chen et al., 2012	Bone	Decellularized + Dried	Single membrane	Human dental apical papilla cells	Basement membrane or Stromal	In vitro/Ex vivo
Semyari et al., 2015.	Bone	Fresh decellularized rabbit amnion	Single membrane/Cover	Rabbit adipose-derived MSC	NS	In vitro/Ex vivo + In vivo
Akazawa et al., 2016	Bone	Cryopreserved + Decellularized	Single membrane/Cover	Human calvaria osteoblasts Human dermal fibroblasts Human umbilical vein endothelial cells Mouse osteoblasts Human periodontal ligament stem cells	NS	In vitro/Ex vivo + In vivo
Tang et al., 2017	Bone	Fresh + De-epithelialized	Single membrane	Human umbilical vein endothelial cells Rat bone marrow MSC	NS	In vitro/Ex vivo
Akhlaghi et al., 2019	Bone	Decellularized + Lyophilized	Single membrane/Cover	Buccal fat pad-derived stem cells	NS	Clinical study
Ahn et al., 2006	Oral mucosa	De-epithelialized + Lyophilized	Single membrane/Cover	Rabbit oral keratinocytes	Basement membrane	In vitro/Ex vivo + In vivo
Amemiya et al., 2010	Oral mucosa	Cryopreserved + De-epithelialized	Single membrane/Cover	Human oral mucosal epithelial cells	Basement membrane	In vitro/Ex vivo + In vivo
Amemiya et al., 2009/2015	Oral mucosa	Cryopreserved + De-epithelialized	Single membrane/Cover	Human oral mucosal epithelial cells	Basement membrane	Clinical study
Hsueh et al., 2016	Oral mucosa	De-epithelialized + air dried	Single membrane	Human oral mucosal epithelial cells	Basement membrane	In vitro/Ex vivo
Amemiya et al., 2008	Periodontal	Cryopreserved + De-epithelialized	Single membrane/Cover	Dog periodontal ligament cells	Basement membrane	In vivo
Iwasaki et al., 2013	Periodontal	Decellularized + Cryopreserved	Single membrane/Cover	Human periodontal ligament stem cells	NS	In vitro/Ex vivo + In vivo
Amemiya et al., 2014	Periodontal	De-epithelialized	Single membrane/Cover	Human periosteum derived stem cells	NS	In vitro/Ex vivo + In vivo
Wu et al., 2015	Periodontal	De-epithelialized	Single membrane/Cover	Human adipose-derived MSC	Basement membrane	In vitro/Ex vivo + In vivo
Honjo et al., 2015	Periodontal	Cryopreserved + De-epithelialized	Amnion placed on a cell culture insert	dental pulp-derived cell sheet	Basement membrane	In vitro/Ex vivo
Zhang et al., 2006	Nerve	NS in the abstract/Not translated to English	A scroll/wrap of amnion	Autogenous Schwann cell	NS in the abstract/Not translated to English	In vivo
Li et al., 2013	Nerve	Fresh	A scroll/wrap of amnion	Allogenic human umbilical cord MSC	NS	Clinical study
He et al., 2002	Tendon	De-epithelialized + Cryopreserved	A scroll/wrap of amnion	Fetal rabbit skin fibroblasts	Attachment on ECM and proliferation on stromal layer	In vitro/Ex vivo + In vivo
Parveen et al., 2019	Cardiac	Trypsinized + Cryopreserved	Single membrane	Human-induced pluripotent stem cell-derived cardiomyocytes	Basement membrane (?)	In vitro/Ex vivo

ECM = Extracellular Matrix; MSC = Mesenchymal Stromal Cells; NS = Not Specified; ?: information assumed by the authors from article content; De-epithelialized = amnion without AEC; Decellularized = amnion without AEC and AMSC.

## Data Availability

Not applicable.
